# Designing mesoporous Cu_2_SnS_3_ on nickel foam as a self-supported hydrogen evolution catalyst in a neutral electrolyte

**DOI:** 10.1039/d6na00853d

**Published:** 2026-10-21

**Authors:** Ritika Sharma, Bhaskar Chandra Mohanty

**Affiliations:** a Department of Physics and Materials Science, Thapar Institute of Engineering and Technology Patiala 147004 India bhaskar@thapar.edu

## Abstract

Although electrochemical water splitting has emerged as an attractive technique for hydrogen generation, high cost and scarcity of noble-metal catalysts remain a concern. While neutral electrolytes offer advantages in stability and reduced corrosion, they suffer from poor reaction kinetics compared to acidic media. Therefore, designing low-cost electrocatalysts for the hydrogen evolution reaction (HER) that can efficiently operate in neutral electrolytes is an important process step in this technology. Herein, we report the successful synthesis of earth-abundant mesoporous cubic Cu_2_SnS_3_ (CTS) nanoparticles and demonstrate enhanced electrochemical activity of these nanoparticles supported on a conductive nickel foam scaffold in a neutral electrolyte. The engineered mesoporosity significantly enhances the density of accessible catalytic sites, while the Ni foam facilitates rapid charge transfer. Electrochemical measurements reveal a low overpotential of ∼288 mV, a Tafel slope of ∼329 mV dec^−1^, an exchange current density of 1.46 mA cm^−2^ and a low charge transfer resistance of ∼301 Ω. These results highlight size-regulated CTS nanoparticles as a cost-effective and efficient solution for hydrogen generation under environment friendly, neutral pH conditions.

## Abstract 2

With earth-abundant composition, mesostructured morphology, favourable electrode–electrolyte interface and low charge transfer resistance, our results establish Cu_2_SnS_3_ as a promising non-noble metal electrocatalysts for HER under neutral conditions.

## Graphical abstract

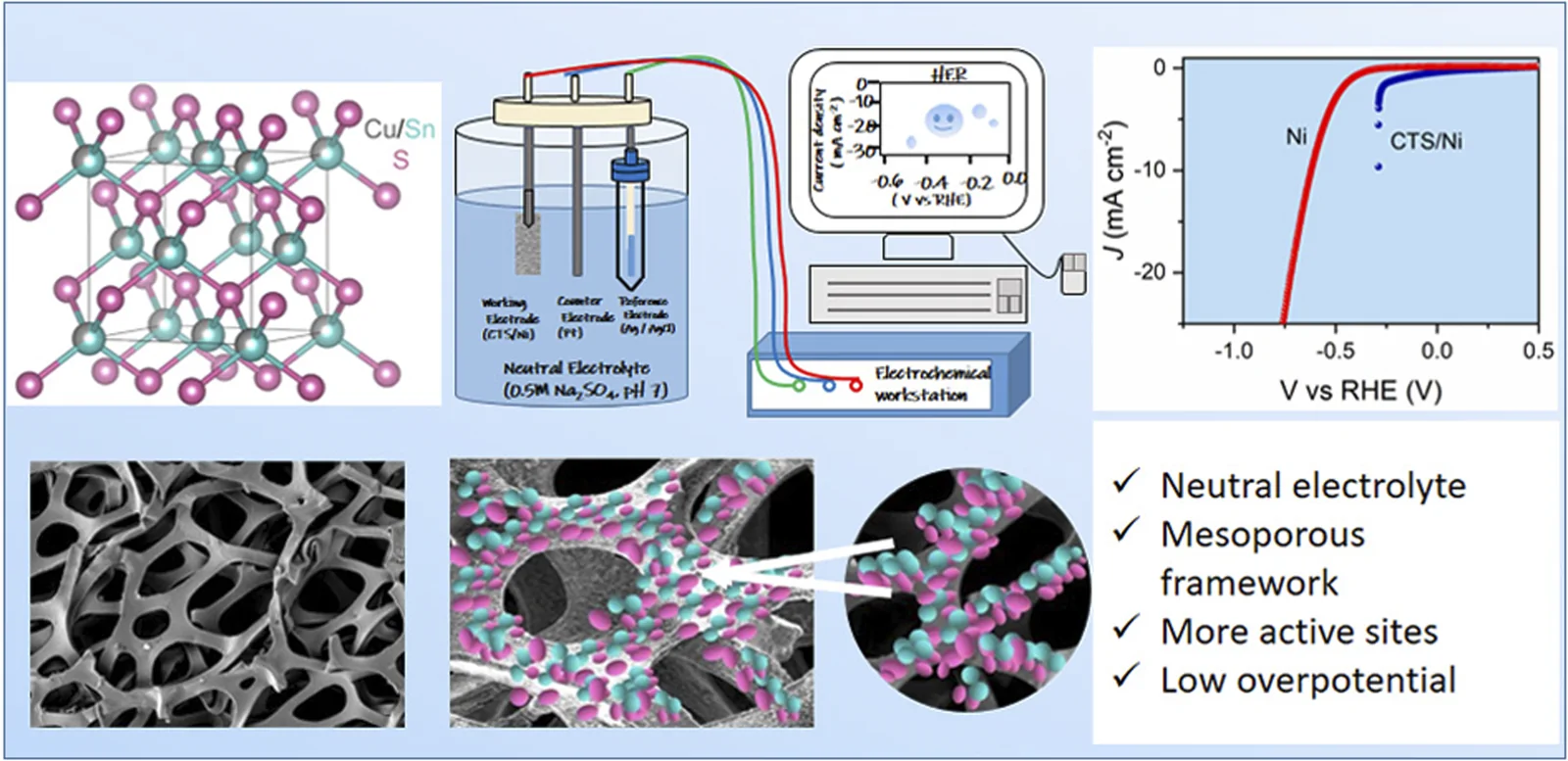

## Introduction

In the quest for carbon-neutral energy, hydrogen generation has evolved as a potential technology for sustainable development.^1–5^ Among the various strategies explored for hydrogen generation, electrochemical water splitting stands out.^2,6^ However, the viability of this approach depends heavily on the development of electrocatalysts that exhibit low overpotentials, high intrinsic activity, fast reaction kinetics, and robust long-term stability. While noble-metal-based materials (*e.g.*, Pt and Pt-group metals) possess superior catalytic activity with near-zero overpotentials,^7,8^ their scarcity and high cost severely limit large-scale implementation. Consequently, significant research has focused on earth-abundant alternatives, including transition-metal sulfides, phosphides, carbides, nitrides, selenides, and oxides.^9–17^ Among these, metal chalcogenides are particularly promising because of their metal–sulfur coordination, electronic structure, surface morphology and defect chemistry, and they can be tailored to regulate the adsorption thermodynamics of reaction intermediates and enhance interfacial electron-transfer processes.

Despite remarkable advances in HER electrocatalysis, a large fraction of high activity in non-noble metal catalysts has been observed under strongly acidic or alkaline conditions. Such operating conditions impose additional requirements on the catalyst related to corrosion resistance, system durability and electrolyte handling. Conversely, the HER in neutral electrolytes offers an environmentally benign and sustainable pathway. However, neutral-medium HER is intrinsically more challenging.^18–21^ The limited availability of H^+^ relative to acidic electrolytes requires considerable involvement of water molecules (H_2_O + e^−^ → H* + OH^−^) as the proton source. Thus, interfacial water dissociation, proton transport, hydrogen-intermediate adsorption and charge transfer collectively create additional kinetic barriers.^18–21^ Designing cost-effective catalytic surfaces capable of driving efficient HER under neutral conditions therefore continues to pose an important material challenge.

Ternary copper–tin–sulfur compounds offer a suitable material platform to address this challenge.^9,11–17^ In particular, Cu_2_SnS_3_ (CTS) an earth-abundant, inexpensive chalcogenide possesses a chemically versatile ternary sulfide framework. Compared with conventional binary sulfides, the coexistence of Cu and Sn cations coordinated with S can generate heterogeneous local electronic environments that may facilitate surface charge redistribution and adsorption of reaction intermediates. Importantly, previous studies have demonstrated the utility of CTS in various electrochemical applications, including counter-electrodes and acidic HER.^22–24^ However, its electrocatalytic capabilities under neutral conditions remain largely unexplored. The existing studies further highlight that its HER activity is highly sensitive to morphology, composition, surface site accessibility, and electronic conductivity.^25–28^ For instance, recent studies have demonstrated the microstructural variation in CTS enhanced electrocatalytic performance under acidic conditions.^24^ Xu *et al.* reported that combining CTS with reduced graphene oxide significantly boosted HER activity in acidic media by exposing active sites and lowering charge-transfer resistance.^29^ Although these observations support CTS as a promising HER electrocatalyst, whether CTS can sustain efficient HER in neutral media without relying on noble metals remains mostly unexplored. This question is particularly significant because the mechanistic requirements of neutral-medium HER deviate substantially from those prevailing in acidic electrolytes as mentioned earlier. Also, a catalyst operating in this regime must simultaneously provide accessible interfacial sites, favourable catalyst–electrolyte interaction, sufficient electronic transport and efficient transfer of charge toward adsorbed hydrogen-containing intermediates. Achieving these characteristics would broaden the applicability of the Cu–Sn–S materials.

Herein, we demonstrate mesostructured CTS as a noble-metal-free HER electrocatalyst in neutral 0.5 M Na_2_SO_4_ electrolyte. Despite the kinetically demanding neutral medium the CTS catalyst supported on a three-dimensional Ni-foam scaffold delivers an overpotential of ∼288 mV *vs.* RHE at −10 mA cm^−2^, demonstrating appreciable HER activity. The obtained Tafel slope of ∼329 mV dec^−1^ indicates that the reaction remains kinetically constrained, while the promising overpotential demonstrates that CTS provides catalytically accessible sites capable of sustaining the HER without the need for a strongly acidic or alkaline electrolyte. Furthermore, to correlate the catalytic behaviour with electrolyte-accessible surface sites and interfacial charge transfer, the electrochemically active surface area and wettability analyses are employed. Highlighting its earth-abundant composition, mesostructured morphology, favourable electrode–electrolyte interface and a low charge transfer resistance of ∼301 Ω, the results establish CTS as a promising platform for further development of non-noble metal electrocatalysts for hydrogen production under neutral conditions.

## Experimental details

A precursor solution was prepared by dissolving copper(ii) chloride dihydrate (CuCl_2_·2H_2_O, ≥99%, Sigma Aldrich), stannous chloride dihydrate (SnCl_2_·2H_2_O, 98%, AR/ACS Loba Chemie), thiourea (CH_4_N_2_S, 99%, AR Loba Chemie) in appropriate amounts at a fixed stoichiometric ratio (Cu : Sn : S = 2 : 1 : 3) in 20 mL of 2-methoxyethanol under constant magnetic stirring for 2 hours at 50 °C. A clear transparent pale yellow solution was obtained, which was then transferred into a 50 mL Teflon-lined stainless steel autoclave. The autoclave was sealed and maintained at 200 °C for 24 hours. Preliminary optimization experiments identified 200 °C and 24 h as the optimal parameters within the evaluated ranges (100–200 °C and 8–24 h). Powder X-ray diffraction and Raman spectroscopy (presented in the following section) confirmed that these conditions produced well-crystallized, phase-pure CTS nanoparticles without requiring post-synthesis annealing. Lower temperatures or shorter reaction times resulted in secondary phases and incomplete crystallization. After the reaction, the autoclave was allowed to cool naturally to room temperature. The resulting black precipitate was collected by centrifugation, washed several times with deionized water to remove residual impurities, and dried at 80 °C for 8 hours. The process steps for the synthesis of the nanoparticles are summarized in Fig. 1. In order to examine the influence of precursor concentration on phase formation and structural integrity, a series of solutions with varying molarities of Cu (0.25 M, 0.5 M, and 0.75 M) were prepared, while maintaining a constant Cu : Sn : S (2 : 1 : 3) stoichiometric ratio and identical hydrothermal synthesis conditions. Notably, the variation in precursor molarity did not result in any observable change in the phase as evaluated from the X-ray diffraction (XRD) measurements. Furthermore, the average crystallite size estimated from the FWHM of the most intense peaks of the XRD patterns remained nearly the same. The sample prepared with the lowest molarity (0.25 M) was used consistently across all the subsequent investigations.

**Fig. 1:**
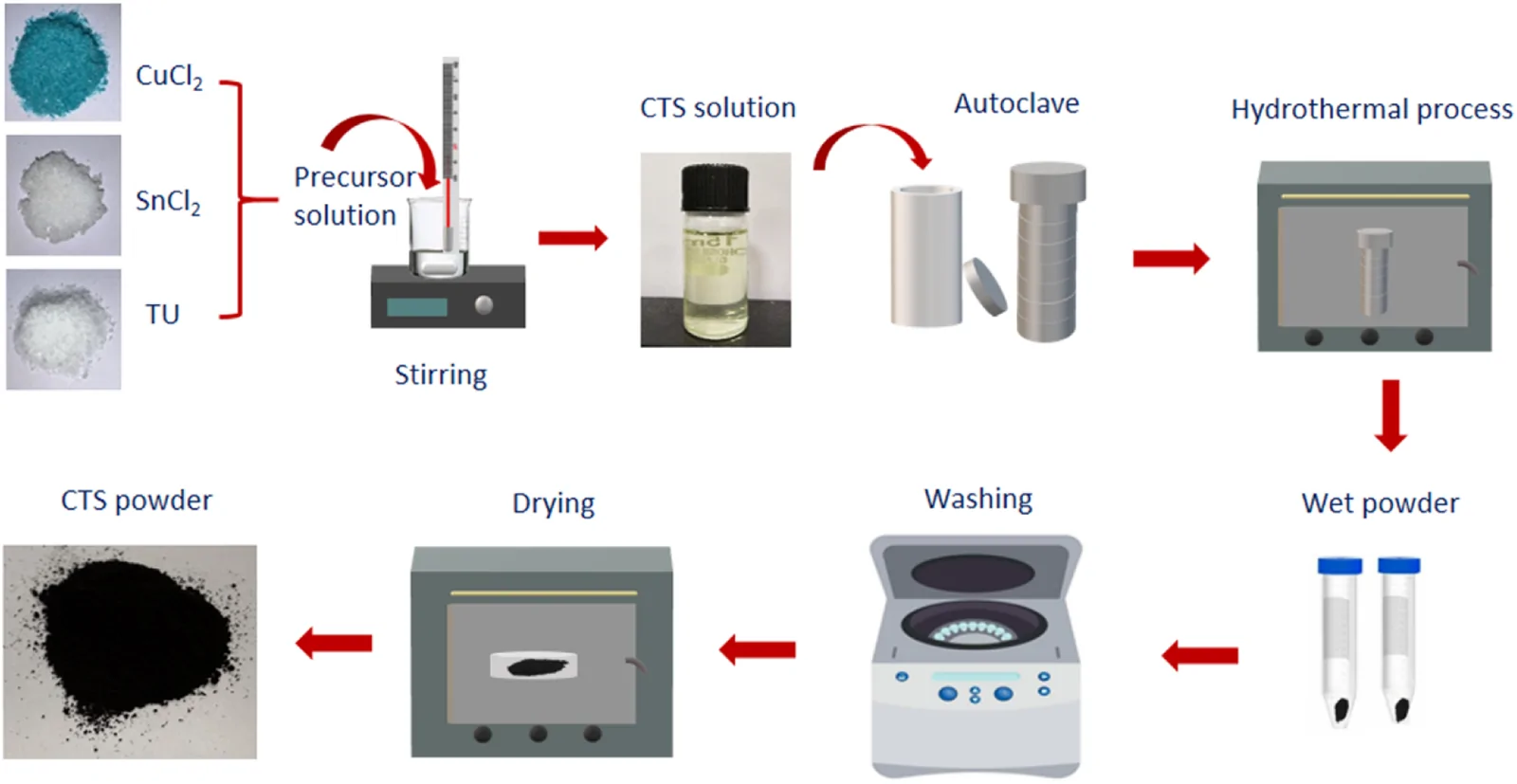
Schematic illustration of the stepwise synthesis process of CTS nanoparticles *via* the hydrothermal route at 200 °C for 24 hours.

The synthesised CTS powder was investigated by XRD using a Rigaku SmartLab SE diffractometer with Cu K_α_ radiation. A Carl Zeiss Sigma 500 field emission scanning electron microscope (FESEM) was used to study the surface morphology. Microstructure of the particles was examined by high resolution transmission electron microscopy (HRTEM) using a Themis 300 (Thermo Scientific) double corrected system. Raman spectroscopy data were acquired on a LabRAM HR Evolution Raman spectrometer (HORIBA Scientific) using a He–Ne laser source with an excitation wavelength of 532 nm. X-ray photoelectron spectroscopy (XPS) measurements were performed using an ESCALAB Xi + system (Thermo Scientific). The XPS measurements were carried out with Al K_α_ X-ray radiation (1486.6 eV). All measurements were conducted at room temperature, with energy resolution of about 0.8 eV. The Brunauer–Emmett–Teller (BET) measurements were carried out using a Microtrac Belsorp Mini-II surface analyser (Bel Japan, Inc.). The contact angle measurement was carried out on a contact angle goniometer (Apex Technologies) to evaluate the wettability of the prepared electrode at room temperature with a 3 µL droplet of 0.5 M Na_2_SO_4_ electrolyte. The electrochemical measurements, impedance spectroscopy (Nyquist and Bode phase plots), cyclic voltammetry (CV), linear sweep voltammetry (LSV) and chronoamperometry were carried out using an electrochemical workstation (Bio-logic EC lab instrument, SP-300) in the standard three-electrode configuration under dark/non-illuminated conditions. A digital photograph of the setup used in this study is shown in Fig. S1 (SI). The working electrode was prepared by drop-casting a well-dispersed CTS nanoparticle NMP suspension onto a nickel foam substrate followed by drying to ensure good mechanical adhesion. Pt was employed as the counter electrode, while an Ag/AgCl electrode was used as the reference electrode. A note of caution here is that Pt dissolution and its subsequent redeposition onto the working electrode can artificially enhance apparent HER activity. Therefore, a graphite rod can be a safer alternative. However, as presented in the following sections, the likelihood of significant Pt interference is minimum in our study. First, the time duration was short enough for severe Pt accumulation. Although chronoamperometry measurements were carried out for 24 hours, the current remained very steady. Second, we performed identical control measurements on bare Ni foam using the same Pt counter electrode. The CTS/Ni foam exhibited better performance compared to the bare Ni foam under the exact same conditions, which confirms that the electrocatalytic activity originates predominantly from the CTS catalyst. The electrolyte was prepared using 0.5 M Na_2_SO_4_ with a stabilized pH of 7.0. The electrochemical potentials were recorded with respect to the Ag/AgCl reference electrode and converted to the reversible hydrogen electrode (RHE) scale. All measurements were conducted under ambient atmospheric conditions at room temperature (∼25 °C) in a neutral electrolyte (0.5 M Na_2_SO_4_, pH 7). Baseline substrate controls were recorded under identical conditions. It may be mentioned that in the absence of continuous inert gas purging, dissolved oxygen in the electrolyte might interfere with the results. However, as discussed in the following sections, baseline tests on bare Ni foam alongside literature precedence and experimental conditions (pH, potential window, *etc.*) suggest that the overall conclusions are not significantly affected by the dissolved oxygen.

## Results and discussion

Nanoparticles of various binary and ternary semiconductors with well-defined shapes and uniform sizes have been synthesized at relatively low temperatures using the hydrothermal process. The high pressure in the autoclave reportedly facilitates breakdown and reaction of the reagents leading to the formation of the desired phase.^30^ In the present case, the nominal reaction temperature of 200 °C is found to be adequate enough for the formation of the CTS phase. A typical XRD pattern of the obtained powder (Fig. 2a) exhibits several distinct diffraction peaks, indicating the polycrystalline nature of the sample. The peaks observed at 2*θ* values of 28.4, 32.93, 47.30, 56.09, 69.22, 76.45° and 88.0° were identified with the (111), (200), (220), (311), (400), (331) and (422) crystal planes of cubic CTS (ICDD no. 01-089-2877), respectively. The higher intensity of the peak corresponding to the (111) plane indicates the preferential crystallite growth direction, a frequently observed characteristic feature in cubic CTS.^22,31^ Rietveld refinement was performed to validate the structural details on the XRD data using the FULLPROOF suite.^32^ An asymmetric pseudo-Voigt function was used to model the diffraction peak shapes. The background intensity of each profile was refined with a six-coefficient polynomial. The refinement was carried out iteratively till a convergence was reached. Fig. 2a shows the refined pattern that matches well with the experimental data with a *χ*^2^ value (goodness of the fit) of 5.44, and *R*_Bragg_ and *R*_F_ of 9.586 and 11.73, respectively. The obtained *R*-factor suggests reasonable agreement between the observed and calculated peak intensities.^33,34^ However, the elevated profile reliability factors and visible residual features around the strongest peak intensity suggest that the refinement is limited by the possible preferred orientation.^33,34^ Nevertheless, the close correspondence of the observed and calculated peak positions verifies the cubic CTS phase (space group of *F*4̄3*m* with a lattice constant 5.4352 Å). The crystal structure obtained from the refinement is shown in Fig. 2b. The bond length of (Cu/Sn)–S and the bond angle S–(Cu/Sn)–S are found to be 2.3513 Å and 109.4712°, respectively. The formation of the cubic CTS phase was further supported from the analysis of the Raman spectrum of the sample. A representative Raman spectrum of the powder exhibits superimposed peaks at about 290, 313 and 332 cm^−1^ (Fig. 2c), which are indicative of the formation of the cubic CTS phase, and are consistent with the previous reports.^35–38^

**Fig. 2:**
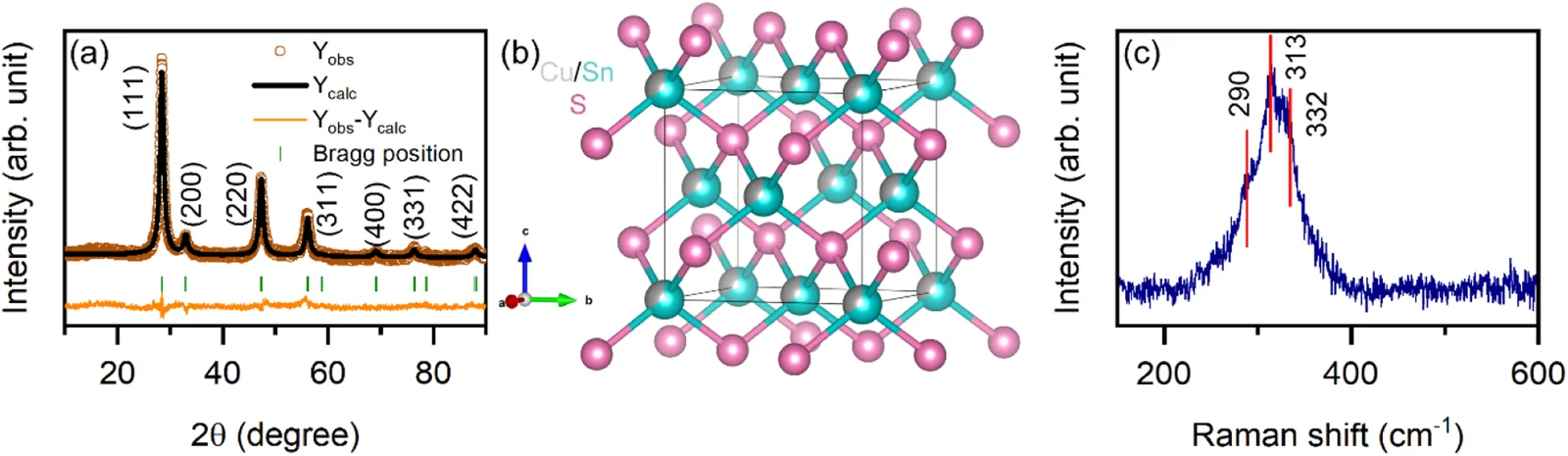
(a) Rietveld refinement of the typical XRD pattern confirming phase purity for cubic CTS nanoparticles (ICDD no. 01-089-2877); (b) crystal structure for cubic CTS nanoparticles; (c) Raman spectrum confirming characteristic vibrational modes associated with cubic CTS.

The details of the morphological and elemental characterization of the synthesized CTS nanoparticles supported on the Ni foam are presented in Fig. 3. Fig. 3a–c show FESEM images at increasing magnifications. At low magnification (Fig. 3a), a highly porous, interconnected three-dimensional Ni foam network is visible, providing a high surface area and open channels for mass transport. Fig. 3b reveals the uniform distribution of CTS nanoparticles decorating the struts of this foam, while the high-magnification view in Fig. 3c highlights that these deposits consist of densely packed, spherical sub-micron clusters. The low-magnification TEM image (Fig. 3d) shows an agglomeration of small nanoparticles. Fig. 3f highlights these very small nanostructures with red outlines, demonstrating that the larger clusters observed in the SEM image are actually aggregates of fine nanocrystals. The Selected Area Electron Diffraction (SAED) pattern shown in Fig. 3e displays well-defined concentric rings, which is typical of a highly crystalline but randomly oriented polycrystalline material. The diffraction rings are indexed to the (111), (220), and (311) planes of the cubic CTS phase, matching well with the results of the XRD measurement (Fig. 2). Furthermore, the HRTEM image in Fig. 3g exhibits clear, parallel atomic lattice fringes. The interplanar *d*-spacing is measured to be ∼0.315 nm, which corresponds to the dominant (111) lattice plane of the crystal phase.

**Fig. 3:**
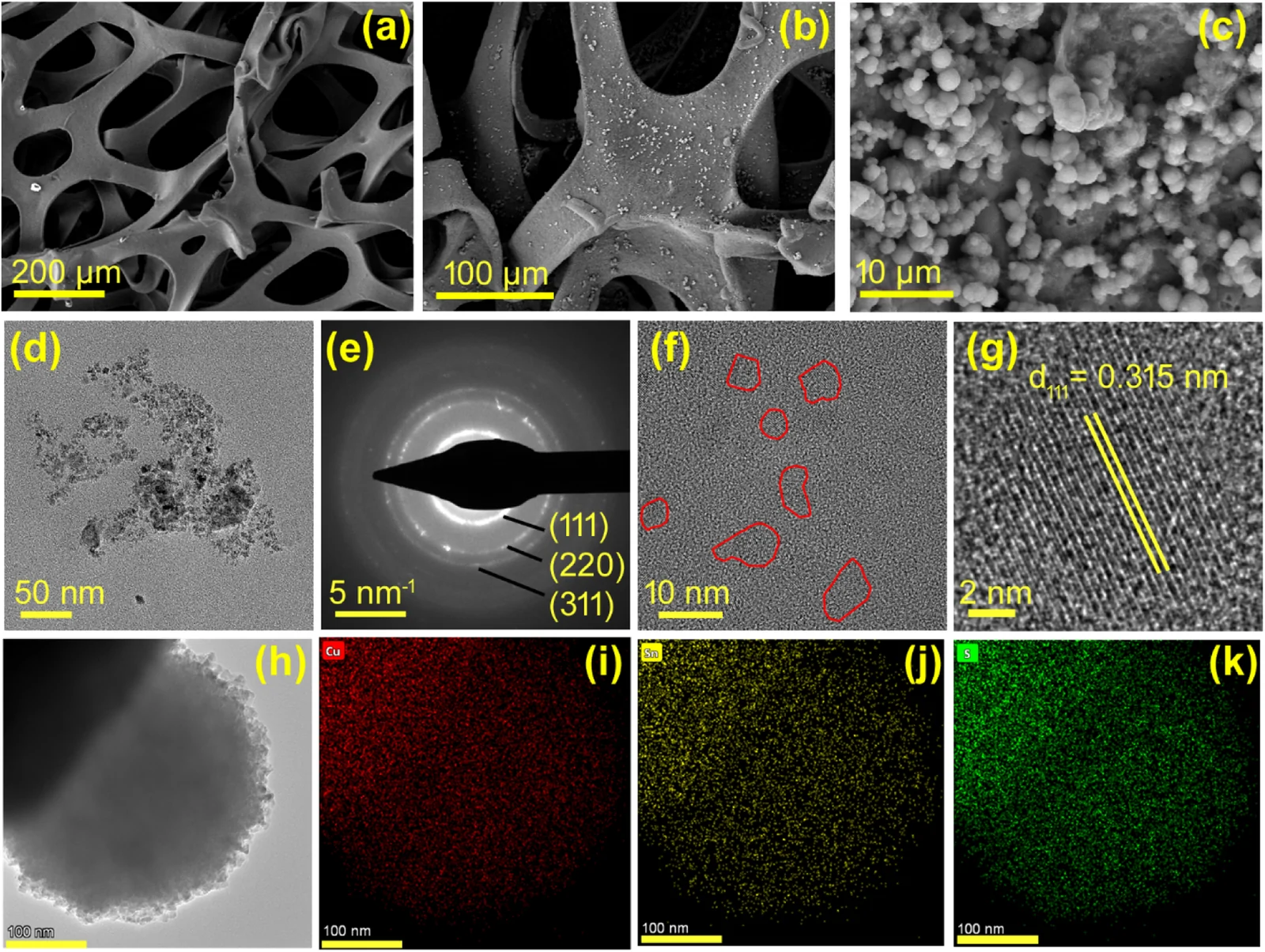
(a) FESEM micrographs of Ni foam; low (b) and high (c) magnification images of CTS nanoparticles supported on Ni foam; (d) TEM image of CTS nanoparticles; (e) SAED pattern with indexed *hkl* planes of cubic CTS; (f) multiple fringe patterns corresponding to CTS nanostructures outlined in red; (g) lattice fringe pattern corresponding to the (111) plane of cubic CTS; (h)–(k) HAADF-STEM images of the constituent elements of CTS nanoparticles.

To confirm the chemical homogeneity and phase purity of the synthesized multi-component nanostructures, the EDS elemental mapping was performed in conjunction with High-Angle Annular Dark-Field Scanning Transmission Electron Microscopy (HAADF-STEM). The elemental maps (Fig. 3i–k) show a highly uniform and overlapping distribution of Cu, Sn and S across the nanostructure. The lack of localized element-rich clusters or phase segregation confirms that a single-phase ternary CTS compound was successfully synthesized.

The chemical state of the elements in the nanoparticles was examined using XPS measurements. Such an assessment is very important as the electrochemical reactions are sensitive to electronic state and the coordination environment, especially those of copper. For example, for Cu^1+^, the d-band centre is located sufficiently below the Fermi level that helps to avoid a large hydrogen chemisorption without compromising efficient electron transfer to adsorbed protons. Contrastingly, Cu^2+^ introduces states that can act as deep recombination centres.^39^ Liu *et al.* demonstrated that by tuning the electronic states of the Cu site, the photocatalytic H_2_ production rate improved significantly.^40^ Similarly, Han *et al.* showed that Cu ions facilitated remarkable H_2_ evolution by regulating Cu states.^41^ The survey scan and the core level spectrum of the elements are shown in Fig. 4a–d. The survey scan (Fig. 4a) shows the presence of peaks corresponding to Cu, Sn, S, C and O. The presence of the C and O is due to the handling of the nanoparticles.^22^ The high resolution core level Cu 2p, Sn 3d and S 2p spectra are presented in Fig. 4b, c and d, respectively. The peaks were deconvoluted using the Gaussian–Lorentzian sum function after Shirley background subtraction using the software XPSPEAK (version 4.1).^42^Fig. 4b shows two distinct peaks at binding energies of 932.5 and 952.3 eV with an energy difference of 19.8 eV. These two peaks correspond to Cu 2p_3/2_ and Cu 2p_1/2_ confirming the Cu^1+^ state in CTS.^22,31^ In Fig. 4c, the two peaks located at 486.4 and 494.9 eV correspond Sn 3d_5/2_ and Sn 3d_3/2_ doublets, respectively, confirming the Sn^4+^ state. The core level 2p spectrum of S is presented in Fig. 4d. The fitting of the experimental data revealed three peaks at about 161.6, 162.8 and 163.6 eV. The peaks at the lowest binding energy values (*i.e.*, at 161.6 and 162.8 eV) correspond to S 2p_3/2_ and S 2p_1/2_, respectively. The integrated peak-area ratio of S 2p_3/2_ to S 2p_1/2_ was approximately 2 : 1, consistent with their respective electronic degeneracies, and the FWHM of the two components was similar. The energy separation between the peaks is about 1.2 eV. As reported in the literature, the peak at 163.6 eV may be associated with surface-related sulfur species, including elemental sulfur or polysulfide-like species, which can arise from residual sulfur-containing species or surface modifications during sample preparation, handling, and exposure to ambient conditions before XPS analysis.^43,44^ Nevertheless, the consistent valence states of Cu^1+^, Sn^4+^ and S^2−^ in the synthesized nanoparticles indicate favourable HER kinetics.^22,31^

**Fig. 4:**
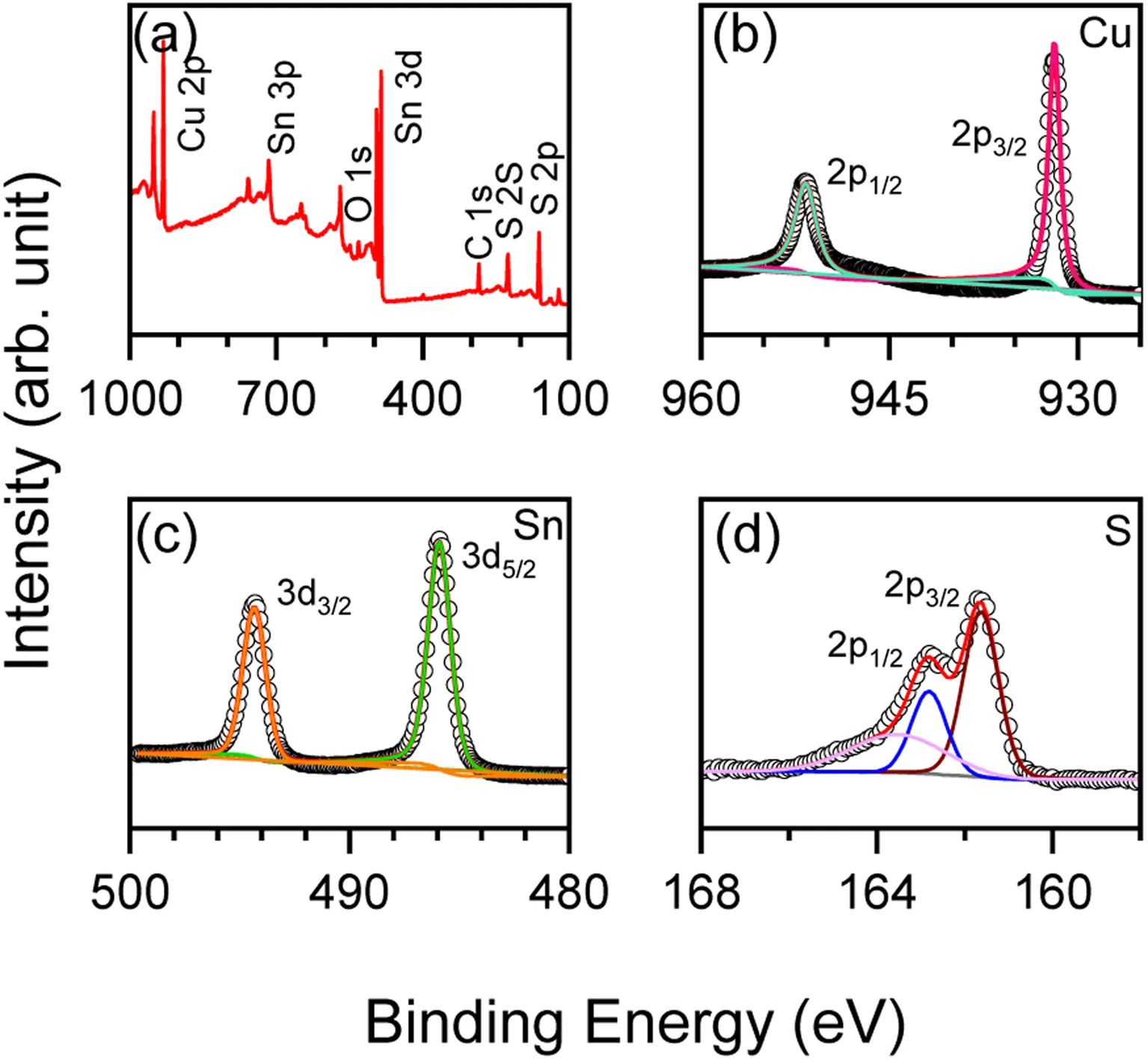
(a) Typical XPS survey scan of the CTS nanoparticles; high-resolution spectra of (b) Cu 2p, (c) Sn 3d, and (d) S 2p states.

The textural properties of a catalyst play a significant role in governing the overall reaction kinetics for the HER.^25–28,31^ A high specific surface area with interconnected pore network substantially enhances the density of electrochemically active sites, and reduces carrier diffusion pathways. This, in turn, helps in increasing the mobility and exchange current density at the interface, as discussed in the following sections. We have carried out the nitrogen adsorption–desorption measurements and the results were analysed with the Barrett–Joyner–Halenda (BJH) model in order to estimate the specific area, pore size and pore volume.^45^Fig. 5a shows a typical nitrogen adsorption–desorption curve obtained for the CTS nanoparticles. For the relative pressure *P*/*P*_o_ = 0.4 to 1.0, a type IV hysteresis loop^46^ is noted from the figure that implies the mesoporous nature of the CTS particles.^46,47^ Typically, a hysteresis in the adsorption–desorption curve is believed to arise from capillary condensation inside the mesopores, indicating the presence of a porous network with interconnected voids instead of isolated pores.^46,47^ The specific surface area of our sample was estimated to be 16 m^2^ g^−1^. The measured BJH pore size distribution is shown in Fig. 5b. The distribution is centred around 10 nm, which quantitatively indicates the mesoporous nature (falling within the interval of 2–50 nm)^31^ of the sample. In view of the small dimension of the nanoparticles directly evidenced from the HRTEM images (Fig. 3d and f), the estimated BJH pore size represents interparticle voids (textural porosity) formed by the random packing and aggregation of the primary nanoparticles, rather than the pores present inside individual CTS nanoparticles. The combination of specific surface area of ∼16 m^2^ g^−1^ along with textural porous nanoparticles of average pore size of 10 nm highlights the nanostructuring obtained during the hydrothermal synthesis of CTS that may offer abundant catalytically active sites. Furthermore, the surface wettability of the electrode was assessed to ensure efficient electrolyte–electrode interaction and the accessibility of electrochemically active sites. The observed angle was ∼18°, which further reduced to ∼14° within 10 ms, evidencing rapid spreading of the electrolyte highlighting the hydrophilic character of the surface (Fig. S2, SI).^48^ This favourable wettability can facilitate penetration of the electrolyte across the porous CTS electrode surface, thereby improving electrolyte–electrode interfacial contact and accessibility of electrochemically active sites.^48,49^

**Fig. 5:**
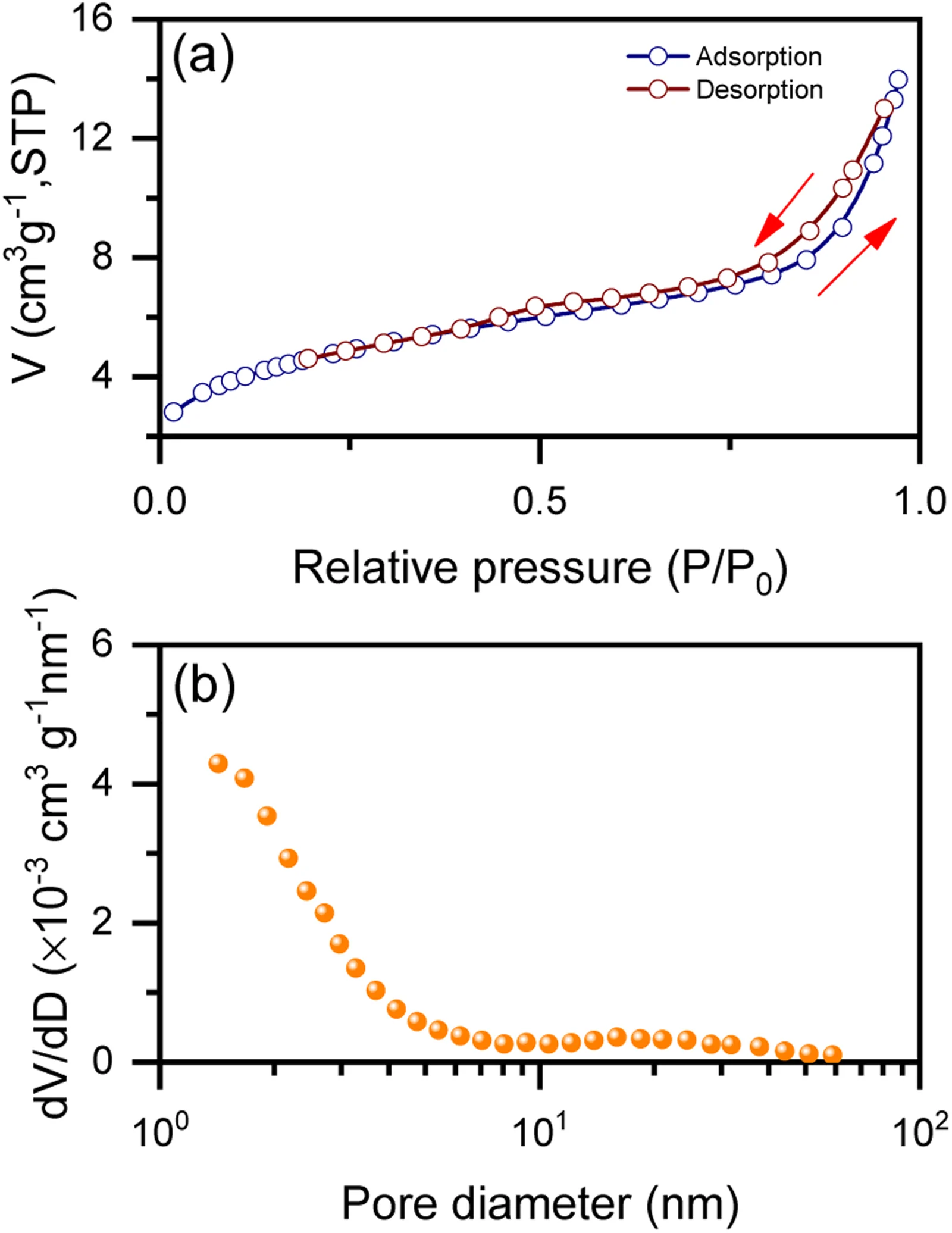
(a) N_2_ adsorption–desorption isotherm of CTS nanoparticles and (b) pore size distribution curve.

The CV measurement was performed at varying scan rates in the potential window −0.3 V to 1.6 V *vs.* RHE as shown in Fig. 6a. The potential window employed for the CV measurements was selected to probe the electrochemical response of the CTS/Ni-foam electrode over a sufficiently broad region while avoiding extensive electrolyte decomposition at the potential limits. The CV curves exhibited broad anodic and cathodic features, which are attributed to reversible or quasi-reversible surface/interfacial redox processes together with electrochemical double-layer charging of the CTS-coated electrode.^50,51^ However, as CTS is supported on Ni foam, a minor contribution from surface redox processes associated with the Ni substrate cannot be completely excluded at sufficiently positive potentials. Therefore, the peaks could not be assigned exclusively to a specific Cu or Sn oxidation-state transition with sufficient certainty. At the more negative potential limit, the rapid increase in cathodic current is associated with the onset of the hydrogen evolution reaction. The current density was found to progressively increase and the redox peaks were systematically shifted with increase in the scan rate. However, at scan rates of 80 and 100 mV s^−1^, the CV curves show considerable overlap. It is believed that at relatively high scan rates, the electrochemical response becomes increasingly limited by ion transport, finite charge-transfer kinetics, and uncompensated resistance, such that the entire porous electrode surface cannot respond uniformly within the shorter experimental timescale.^52,53^ Consequently, increasing the scan rate did not produce a proportional increase in the current density.

**Fig. 6:**
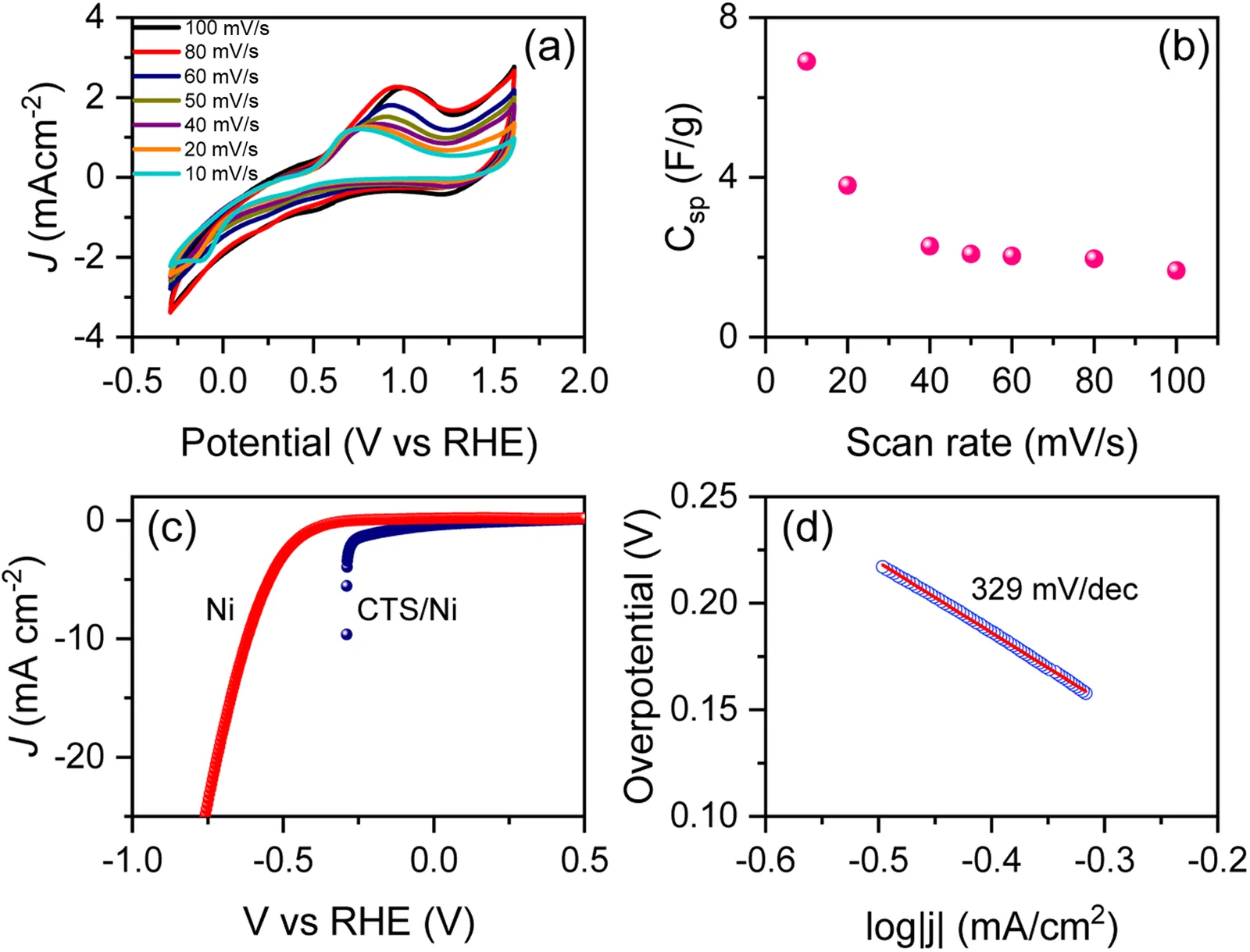
(a) CV curves at varying scan rates; (b) variation of specific capacitance (*C*_sp_) *vs.* scan rate; (c) a representative LSV curve for bare Ni and CTS loaded Ni, and (d) Tafel plot derived from the polarization curve through linear fitting of the selected data region.

Specific capacitance *C*_sp_ for different scan rates was estimated by integrating the corresponding CV curve over the selected potential window according to,1
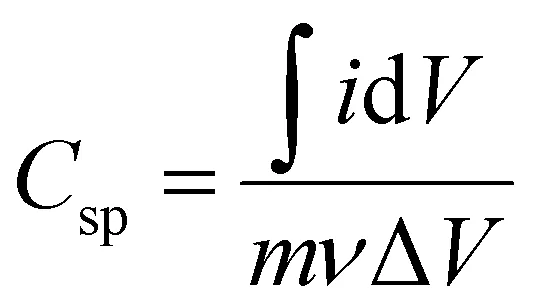
where *i*, *m*, *ν*, and Δ*V* represent the measured current, the mass of the electroactive CTS, the scan rate, and the applied potential window, respectively. The same potential limits and electrode mass were used for all scan rates, and the resulting *C*_sp_ values have been calculated individually. The variation in the calculated *C*_sp_*versus* scan rate is shown in Fig. 6b. With increasing scan rates, the specific capacitance value decreased as a result of poor diffusion of electrolyte ions into the electrode material due to inadequate time.^31^ The electrochemically active surface area (ECSA) of the CTS-coated Ni foam electrode was evaluated *via* double-layer capacitance (*C*_dl_) measurements derived from CV curves at a potential (0.45 V *vs.* RHE, in the present case) in the non-faradaic potential region.^54^ The difference of the capacitive current density, Δ*J* = (*J*_a_ − *J*_c_)/2 (where *J*_a_ and *J*_c_ denote the anodic and cathodic current densities, respectively), was plotted against the scan rate (Fig. S3, SI). The slope of the linear fit yielded a *C*_dl_ value of ∼4.65 mF cm^−2^. Using the relation ECSA = *C*_dl_/*C*_s_ with a literature-standard specific capacitance value of *C*_s_ ≈ 40 µF cm^−2^,^55^ the resulting ECSA is about 116 cm^2^ per 1 cm^2^ of geometric electrode area. The relatively high ECSA is consistent with the porous structure of the CTS framework and indicates a high density of electrolyte-accessible active sites.

Although the HER is thermodynamically favourable at 0 V *vs.* RHE, practical execution requires additional energy to overcome kinetic barriers related to hydrogen adsorption, charge transfer, and interfacial processes. These barriers are quantified using the onset potential (the potential required to reach −1 mA cm^−2^) and the overpotential (the potential required to drive a current density of −10 mA cm^−2^).^20^Fig. 6c shows a typical linear sweep voltammetry (LSV) response of the system. It should be emphasized that the LSV polarization curves have not been subjected to *iR* compensation. Therefore, all following discussions pertaining to the LSV profile and overpotential values are based on these uncorrected curves.

For comparison, the response from the Ni foam substrate (without any CTS loading) is also presented in the figure. Both electrodes displayed low baseline currents at initial potentials, followed by a rapid rise in cathodic current as the potential shifted further negative. However, the CTS/Ni foam exhibited significantly enhanced electrocatalytic performance: bare Ni required a far more negative potential to achieve a current density of −10 mA cm^−2^, whereas CTS/Ni reaches this benchmark at an overpotential of about 288 ± 1 mV. This positive shift confirms that the superior activity originates from the CTS material rather than the substrate. For the CTS/Ni foam, it appears that the number of data points is less in the higher potential region (Fig. 6c). However, this apparent lower density of data points is a natural consequence of the rapid increase in current density. Because the potential is scanned linearly against time, the rapid current rise creates a larger vertical gap between consecutive data points on the plot. It may be noted that since the overpotential was measured directly without any mathematical extrapolation, this larger spacing between data points does not overestimate its value. The CTS/Ni electrode displays a low onset potential of −180 mV (Fig. S4, SI), aligning closely with previously reported values for CTS@rGO (−193 mV).^29^ It is worth mentioning that the obtained value of overpotential (∼288 ± 1 mV *vs.* RHE at −10 mA cm^−2^) is remarkably in good agreement with the reported literature value. Digraskar *et al.* reported an overpotential of 442 mV for CZTS.^56^ For Ni and Fe-based CZTS composites, values of 214 and 300 mV were reported, respectively.^56^ Similarly for Cu_2_FeSnS_4_, reported by Mukurala *et al.*, a high overpotential of 421 mV was obtained.^57^ In the case of CTS, the reported overpotential by Maheshkumar *et al.* and Xu *et al.* is 330 and 337 mV, respectively.^24,29^

Insight into the rate-determining step and catalytic mechanism was obtained from the Tafel analysis of the LSV data. Unlike acidic electrolytes, where protons are easily available, the HER in a neutral solution depends on water reduction, making the primary Volmer step energetically challenging due to the necessity of H–OH bond cleavage.^20^ The polarization response was further examined by using the Tafel equation, *η* = *a* + *b* log|*j*|, where *η* is the overpotential and *j* is the current density.^58^ As detailed in the SI, *η versus* log|*j*| was plotted beyond the HER onset, avoiding the near-equilibrium and transport-affected regions. Fitting of the linear portion of the Tafel plot (Fig. 6d) yielded the Tafel slope of ∼329 ± 1 mV per decade. It suggests that the reaction typically proceeds through the Volmer–Heyrovsky process^20^ and thus, is the rate limiting step. The exchange current density (*j*_o_), was derived from the extrapolation of the Tafel plot to zero overpotential, which serves as a quantitative indicator of the intrinsic catalytic activity to drive the HER under near-equilibrium conditions. In our case, *j*_o_ of ∼1.46 mA cm^−2^ was obtained, which is higher compared to that reported in the literature.^29^ This suggests favourable adsorption and desorption processes even under neutral electrolyte conditions. It may be possible that the stabilized Cu^1+^ assisted in preventing the formation of copper vacancies, thereby reducing the number of compensating defects.^39^ This is reflected in the relatively longer carrier lifetime, as confirmed later in the Bode plot.

The reproducibility of the electrocatalytic performance was evaluated by repeating the HER measurements using three independently prepared CTS/Ni foam electrodes under identical experimental conditions. The corresponding LSV curves showed closely comparable electrocatalytic behaviour characterized by similar values of the onset potential, overpotential and Tafel slope (Table S1, SI). The small variation among the measurements demonstrates good reproducibility of the prepared electrode sample. All performance metrics reported herein reflect the mean values, with uncertainties representing maximum deviation.

To evaluate the electrocatalytic HER activity of the present cocatalyst-free CTS, its performance should be contextualized against the reported Cu-based multinary chalcogenides. However, a direct comparison is challenging because most existing studies were conducted in acidic or alkaline media, whereas this work evaluates activity in a neutral electrolyte. Table 1 summarizes the performance metrics of selected Cu-based ternary chalcogenides alongside the current CTS electrode.^11,24–26,56,57,59–62^ For further context, the limited data available for sulfur-based electrodes in neutral media are compiled in Table S2. Digraskar *et al.* reported an overpotential of 300 mV with a Tafel slope of ∼85 mV dec^−1^ for CZTS.^59^ They attributed it to smaller and homogeneously distributed particle size, which gave more accessible active sites for the HER. On the other hand, Li *et al.* reported a significantly higher overpotential of 561 mV with a Tafel slope of 157.6 mV dec^−1^ for CZTS, reportedly due to the varied contribution of diverse crystal facets towards the electrocatalytic performance.^63^ Maheshkumar *et al.* compared the performance of Cu_2_SnS_3_ and Cu_4_SnS_4_.^24^ They found that the metal to sulphur ratio plays a huge role in the electrocatalytic activity. A high overpotential of 330 and 358 mV with the Tafel slope of 98 mV dec^−1^ and 110 mV dec^−1^ were found for Cu_2_SnS_3_ and Cu_4_SnS_4_, respectively. In a separate study, by varying the morphology of Cu_3_SnS_4_, Maheshkumar *et al.* achieved an improved overpotential of 230 mV with a Tafel slope of 76 mV dec^−1^ due to increased surface area (as a consequence of formation of flower structured nanoplates).^26^ On the other hand, for CuFeS_2_ ultrathin nanosheets with high-index {02̄4} facets, a remarkably low overpotential of 88.7 mV with a Tafel slope of 47 mV dec^−1^ have been reported.^64^ This was attributed to the S^2−^ active sites probably due to desirable Gibbs free energy for atomic hydrogen adsorption. Hu *et al.* again highlighted the importance of morphology and stoichiometry for achieving comparable overpotential to our work.^25^ In this context, the catalytic metrics for the CTS presented in this work especially in a neutral electrolyte with mesoporosity reflect the high electrocatalytic activity with an overpotential of 288 ± 1 mV and a Tafel slope of 329 ± 1 mV dec^−1^. The mesoporous framework not only facilitated the electrolyte penetration as revealed from the contact angle measurement (shown in Fig. S2, SI), but also increased the electrochemically active surface area that helped in effectively mitigating diffusion limitations typically seen in neutral media. It is worth mentioning that comparable (or improved) overpotential and Tafel slope in the present work relative to the existing cocatalyst-free Cu-based multinary systems suggest that the CTS holds high potential within the broader class of electrocatalysts for the HER in the near future.

**Table 1:** Comparison of HER performance metrics of Cu based sulphide catalysts

Catalyst	Electrolyte medium	Overpotential (mV) at −10 mA cm^−2^	Tafel slope (mV dec^−1^)	Reference
CZTS	0.5 M H_2_SO_4_	300	85	59
CZTS	0.5 M KOH	667	—	
CZTS	0.5 M Na_2_SO_4_	690	—	
CZTS	0.5 M H_2_SO_4_	985	155	11
Cu_2_NiSnS_4_		369	76	
Cu_2_MnSnS_4_		705	119	
Cu_2_CoSnS_4_		689	103	
CZTS	0.5 M H_2_SO_4_	442	125	56
Fe-CZTS		300	110	
Ni-CZTS		214	100	
CZTS NCs	0.5 M H_2_SO_4_	561	157.6	63
CZTS PEG	0.5 M H_2_SO_4_	295	133	25
Cu_3_SnS_4_-B	0.5 M H_2_SO_4_	749	127	26
Cu_3_SnS_4_-S	0.5 M H_2_SO_4_	230	76	
Cu_2_SnS_3_	0.5 M H_2_SO_4_	330	98	24
Cu_4_SnS_4_	0.5 M H_2_SO_4_	358	110	
Cu_2_WS_4_	0.5 M H_2_SO_4_	650	121	60
Cu_2_MoS_4_	0.5 M H_2_SO_4_	333	130.3	61
CuGaS_2_/7wt%MoS_2_	0.5 M H_2_SO_4_	464	56.2	62
Cu_2_FeSnS_4_	0.5 M H_2_SO_4_	421	54	57
Cu_2_SnS_3_	0.5 M Na_2_SO_4_	288	329	This work

To assess the frequency-dependent charge transfer and contribution of the mass-transport process, EIS measurements were carried out at an applied potential corresponding to the overpotential (*i.e.*, 288 mV) with a sinusoidal AC voltage perturbation of 10 mV. The Nyquist plot shows an arc spanning the real (*Z*′) and imaginary (–*Z*″) impedance spectrum. The depressed incomplete semicircle demonstrates non-ideal interfacial capacitive nature stemming from the porous electrode, surface homogeneity, defect distribution, *etc.*^65,66^ It was attempted to fit the experimental data using several models. A satisfactory fitting was obtained for the equivalent circuit model shown in the inset. It consists of an uncompensated solution resistance (*R*_1_ ≈ 10^3^ Ω) connected in series with two parallel constant phase element (CPE) combinations, (*R*_2‖_*Q*_2_) and (*R*_3‖_*Q*_3_), representing the porous film properties/charge transfer resistance and interfacial double-layer capacitive behavior, respectively. *R*_2_ the charge transfer resistance was found to be ∼301 Ω. This value is higher than the 73 Ω reported by Xu *et al.* in acidic media.^29^ In contrast, Chen *et al.* recorded a resistance of 341.3 Ω for CTS-S/TiO_2_ in a neutral electrolyte.^67^ For CZTS, Digraskar *et al.* reported a value of 131 Ω, whereas Ni-CZTS and Fe-CZTS exhibited lower values of 98 and 118 Ω, respectively.^56^ Additionally, Mukurala *et al.* observed a value of 320 Ω for the structurally similar material Cu_2_FeSnS_4_.^57^

The corresponding Bode plot (Fig. 7b) displays the phase angle (*ϕ*) and log magnitude (log|*Z*|) as a function of frequency. In the log|*Z*| *vs.* frequency plot, the higher impedance values at very low frequencies are due to contribution of the charge transfer resistance and diffusion-controlled processes. As the frequency increases, a slow decrease in impedance, revealing reduced participation of the above processes, is observed. At high frequencies, the impedance approaches a minimum plateau that is attributed to the intrinsic resistance of CTS electrode and electrolyte. From the phase angle plot, it is found that the phase angle becomes more negative with increasing frequency, touching its maximum negative value (−41°) at around 1–2 Hz. It is because the interfacial response shifts from resistive to capacitive behavior. In the low-frequency region, the charge transfer and diffusion processes play a decisive role, and as the frequency increases, the interfacial relaxation process and the double-layer capacitance begin to dominate, causing the phase angle to shift toward more negative values.^23,65^

**Fig. 7:**
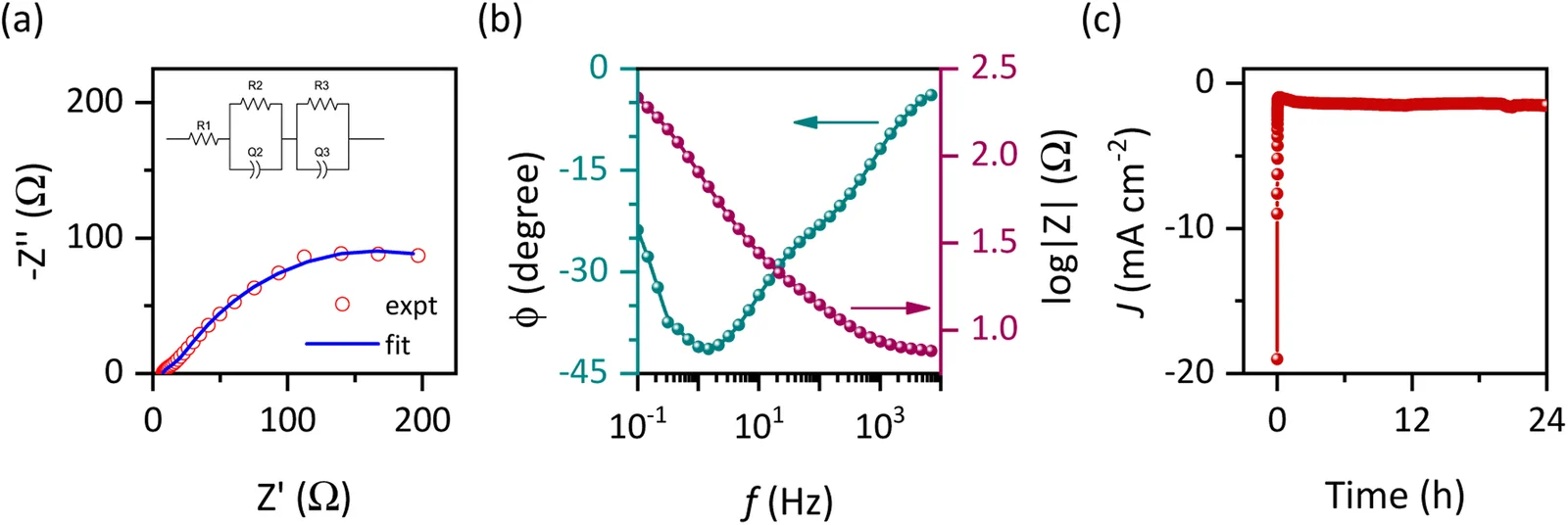
(a) Nyquist plot of CTS nanoparticles obtained at an applied potential of 10 mV; the inset presents the equivalent circuit model; (b) Bode plots (phase angle and impedance magnitude *vs.* frequency); (c) results of the chronoamperometry measurement carried out for 24 hours in a neutral medium.

To assess durability under operating conditions, chronoamperometric measurement was conducted at 288 mV *vs.* RHE, matching the potential of the HER activity. The results depicted in Fig. 7c reveal two zones: an initial transient drop in the current density followed by a steady value of about −1.14 mA cm^−2^. Thus, it is reasonable to state that the reported overpotential of 288 mV at −10 mA cm^−2^ represents the apparent electrocatalytic activity under dynamic LSV polarization conditions rather than a stabilized long-term overpotential. Nevertheless, the observed pronounced drop in the current density toward a quasi-steady-state value has been reported in many studies and has been attributed to several factors including the obstruction of active sites due to accumulation of hydrogen bubbles, increase in local pH that limits mass transport, *etc.*^68–71^ The effects of these factors, however, could not be identified in the present set of experiments. Interestingly, post-measurement characterization revealed no discernible changes in the structural or morphological properties of the catalyst. XRD patterns obtained before and after electrocatalytic testing remained identical (Fig. S5a, SI). XPS measurements before and after the measurements showed similar features except an increase in the intensity of the oxygen-related peak at about 533 eV, as shown in Fig. S5b (SI). This might be due to adsorption of oxygen on to the sample surface during/after the measurement. It may be noted that after removing from the electrolyte (after chronoamperometric measurements of 24 h) the sample was dried in air prior to the XPS measurement. Furthermore, FESEM imaging confirmed that the microstructure was well preserved after measurements (Fig. S5c, SI).

In the present case, the high activity in the neutral electrolyte is thought to stem from a synergistic combination of structural, interfacial, and electronic factors. The mesoporous assembly of nanoparticles provides an accessible surface with abundant exposed catalytic sites, as evidenced by the high double-layer capacitance and corresponding ECSA. Contact angle measurements confirm superior surface wettability, ensuring effective electrolyte infiltration into the porous network. Concurrently, the three-dimensional Ni-foam scaffold acts as a highly conductive current collector that facilitates efficient electron transport, which is further supported by the low charge-transfer resistance observed in EIS measurements. In addition, XPS analysis confirms that copper exists predominantly in the Cu^+^ oxidation state within the CTS lattice. This Cu^+^–S coordination environment likely favours interfacial charge transfer and the binding energetics of hydrogen intermediates (H*). Together, these factors drive the low overpotential and rapid reaction kinetics observed during hydrogen evolution.

## Conclusion

In summary, this work successfully demonstrates the potential of mesoporous cubic Cu_2_SnS_3_ (CTS) nanoparticles as an efficient, noble-metal-free electrocatalyst for hydrogen generation. By addressing the kinetic limitations typically associated with neutral electrolytes, we have shown that microstructural engineering – specifically the creation of a mesoporous framework – combined with a conductive nickel foam scaffold, significantly enhances the hydrogen evolution reaction (HER) characterized by a low overpotential (∼288 mV), favourable Tafel slope (∼329 mV dec^−1^), an exchange current density of 1.46 mA cm^−2^, and a charge transfer resistance of ∼301 Ω. The results imply a durable and cost-effective alternative to scarce platinum-group metals and highlight CTS-based nanostructures as a promising class of catalysts for environmentally friendly neutral-pH water splitting.

## Conflicts of interest

There are no conflicts to declare.

## Supplementary Material

NA-OLF-D6NA00853D-s001

## Data Availability

All the generated data are included in this manuscript and in the supplementary information (SI). Supplementary information: photograph of electrochemical measurement setup; contact angle images; calculation of electrochemically active surface area; determination of onset potential; calculation of Tafel slope and exchange current density; results of stability test; reproducibility results; comparison of the HER electrocatalytic performance of representative chalcogenide-based catalysts reported under neutral electrolyte conditions. See DOI: https://doi.org/10.1039/d6na00853d.
