# Supplementary material for: Designing mesoporous Cu2SnS3 on nickel foam as a self-supported hydrogen evolution catalyst in a neutral electrolyte
Source: Nanoscale Adv. 2026 Oct 21. Online ahead of print. doi: 10.1039/d6na00853d (PMC13746442; doi:10.1039/d6na00853d)
Supplement: Supplementary file 1 [file NA-OLF-D6NA00853D-s001.pdf]

Supplementary Information (SI) for Nanoscale Advances.
This journal is © The Royal Society of Chemistry

**Supplementary Information**

**Designing Mesoporous $Cu_2SnS_3$ on Nickel Foam as a self-supported hydrogen evolution catalyst in a neutral electrolyte**

Ritika Sharma, Bhaskar Chandra Mohanty*
Department of Physics and Materials Science, Thapar Institute of Engineering and Technology, Patiala 147004, INDIA
*Corresponding author: bhaskar@thapar.edu

## 1. Electrochemical measurement setup

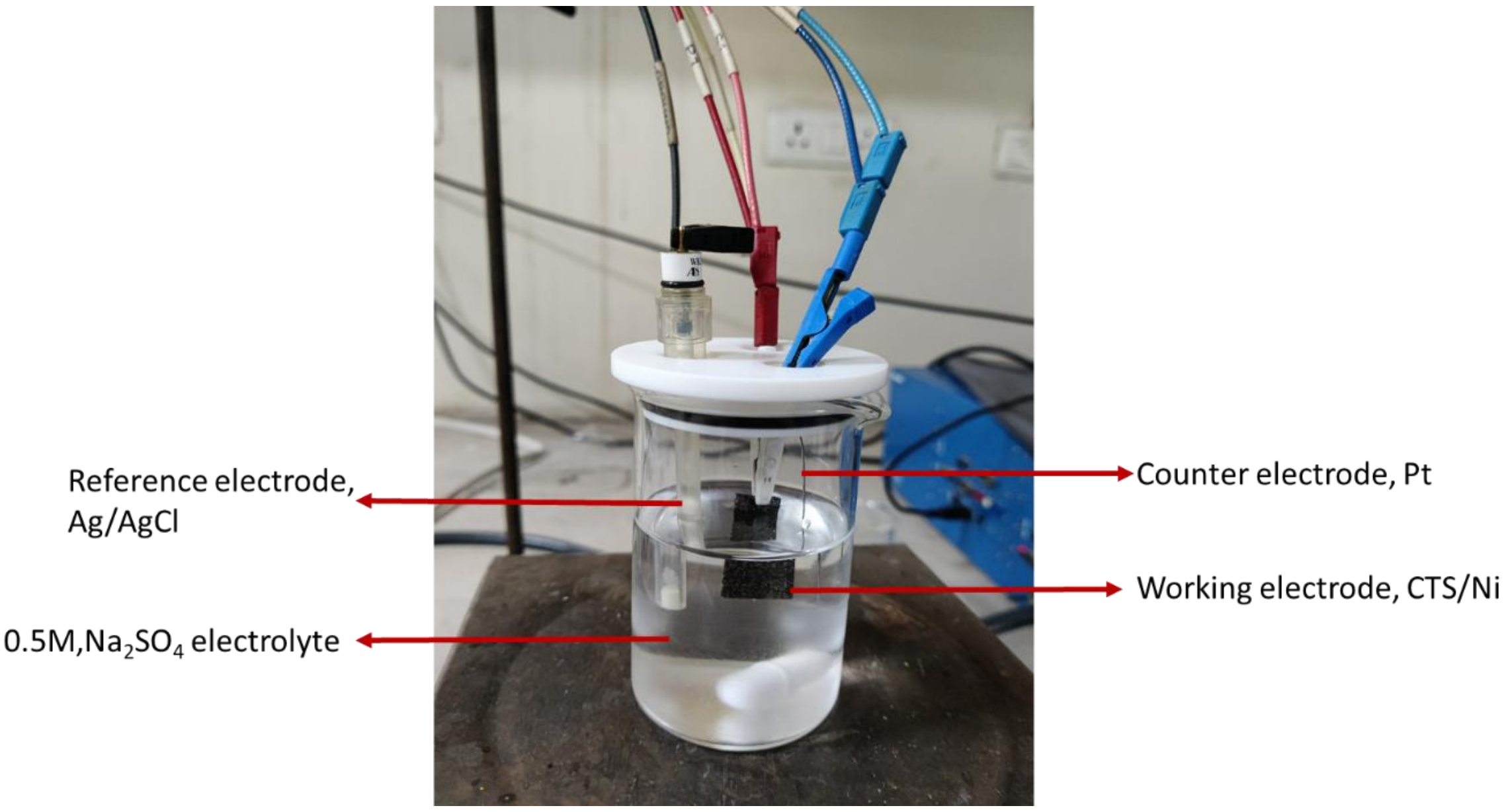


**Fig. S1:** Photograph of the three-electrode electrochemical setup used for electrochemical measurements.

## 2. Contact angle measurement

The contact angle measurement was carried out on contact angle goniometer (Apex Technologies) to evaluate the wettability of the prepared electrode at room temperature.

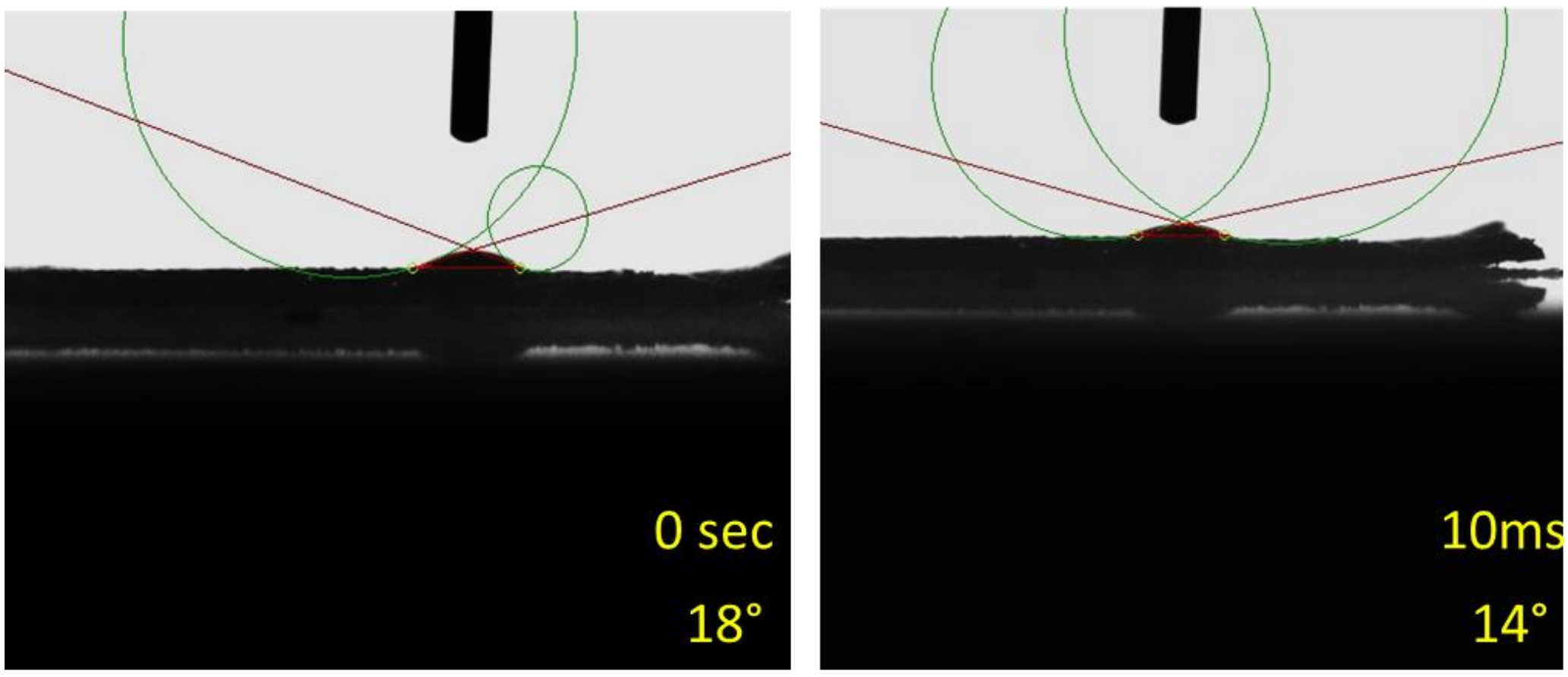


**Fig. S2:** Contact angle images of a_CTS/Ni_electrode at room temperature with a 3 µL droplet of 0.5 M $Na_2SO_4$ electrolyte. The contact angle decreases from approximately 18° at 0 s to 14° after 10 ms.

**3. Calculation of electrochemically active surface area (ECSA)**

The ECSA of the CTS-coated Ni foam electrode was evaluated using the formula

ECSA = $C_{dl}/C_s$, where $C_{dl}$ is the double-layer capacitance obtained from the slope of the capacitive current-density difference (ΔJ) versus scan rate and $C_s$ is the specific capacitance (= 40 µF $cm^{-2}$, as per literature standard). The current-density difference, $\Delta J = (J_a - J_c)/2$ (where $J_a$ and $J_c$ denote the anodic and cathodic current densities, respectively) was determined from CV curves at a potential (0.45 V vs RHE, in the present case) in the non-Faradaic potential region. Figure S3 shows the plot of variation in ΔJ with scan rate.

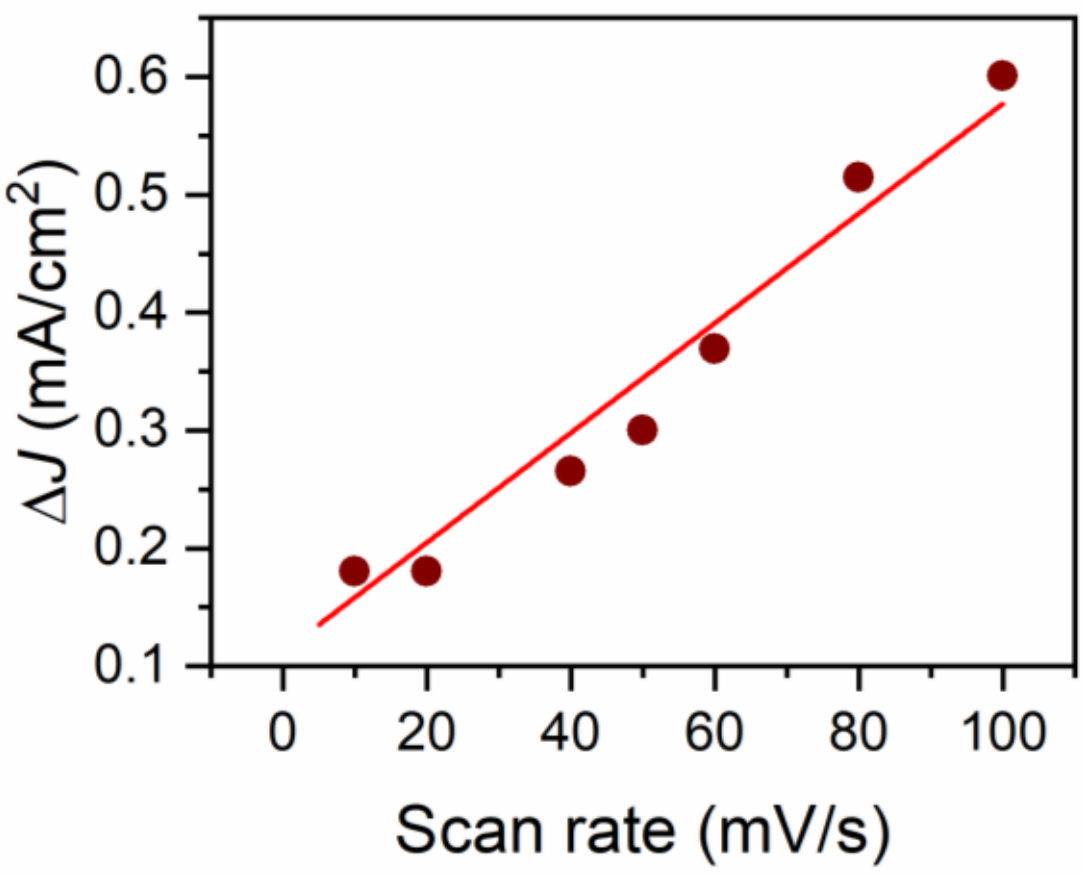


**Fig. S3:** Variation in capacitive current-density difference (ΔJ) with scan rate. Linear fit to the data yielded a slope of 4.65 mF $cm^{-2}$ corresponding to the value of $C_{dl}$.

## 4. Determination of onset potential

The onset potential was calculated as the potential required to drive a current density of -1 mA $cm^{-2}$. Figure S4 shows the full-range LSV and an enlarged HER-relevant potential region to calculate the onset potential. In the present case, an onset potential of -180 mV was obtained, as shown in Fig. S4b.

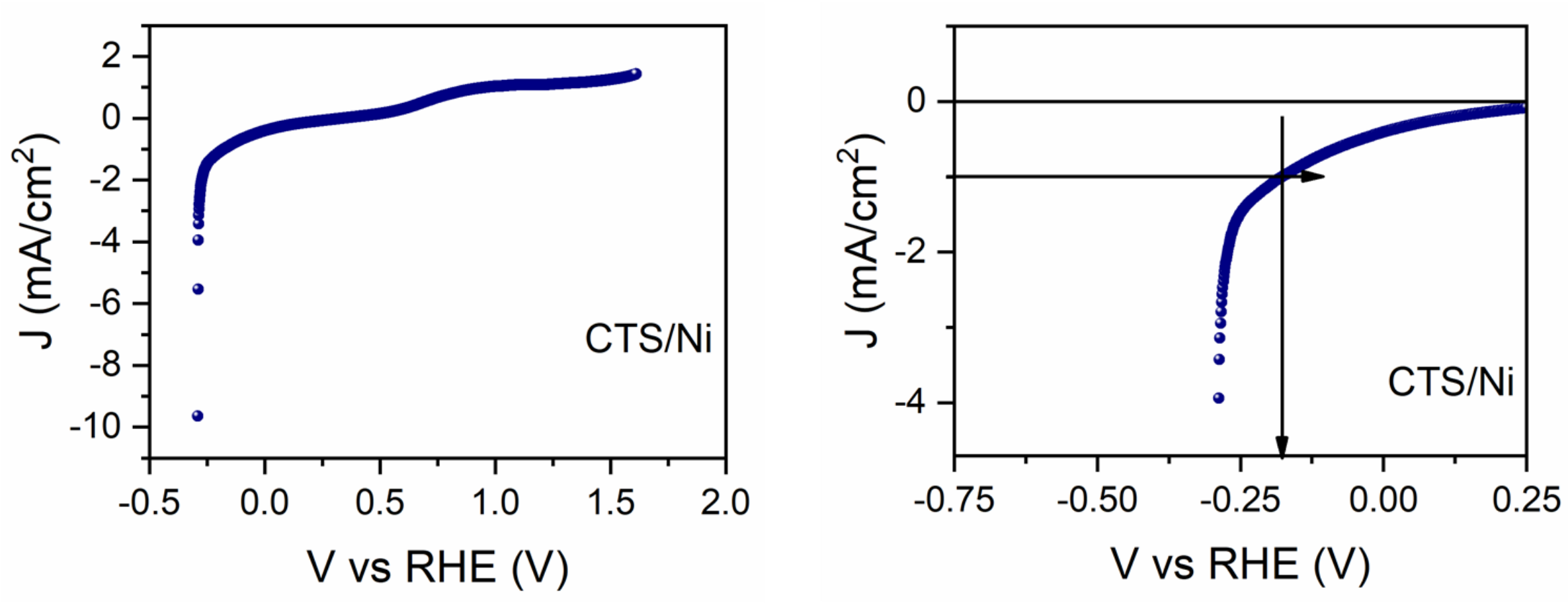


**Fig. S4: (a)** Full-range LSV curve showing the overall electrochemical response of the electrode and **(b)** enlarged HER-relevant potential region for evaluation of the onset potential.

## 5. Calculation of Tafel slope and exchange current density ($j_o$)

The LSV curve for the CTS loaded onto Ni foam (CTS/Ni electrode) is shown in Fig. 6c in the main manuscript. For the calculation of Tafel slope and exchange current density (**$j_o$**)**,** a linear region of the polarization curve was considered, avoiding the near-onset region and the high-current region where mass-transport, bubble formation, or uncompensated resistance effects may influence the response. The linear portion was fitted using the Tafel relation between overpotential (η) and current density (j)

$$\eta = a + b \log|j|$$

where, *a* represents the intercept and *b* denotes the Tafel slope. From the linear regression, the fitted parameters were obtained as *a* = 0.054 V and *b* = -0.329 V $dec^{-1}$.

The exchange current density ($j_o$), was derived from the extrapolation of the Tafel plot to zero overpotential and was found to be ~1.46 mA $cm^{-2}$.

## 6. Stability test

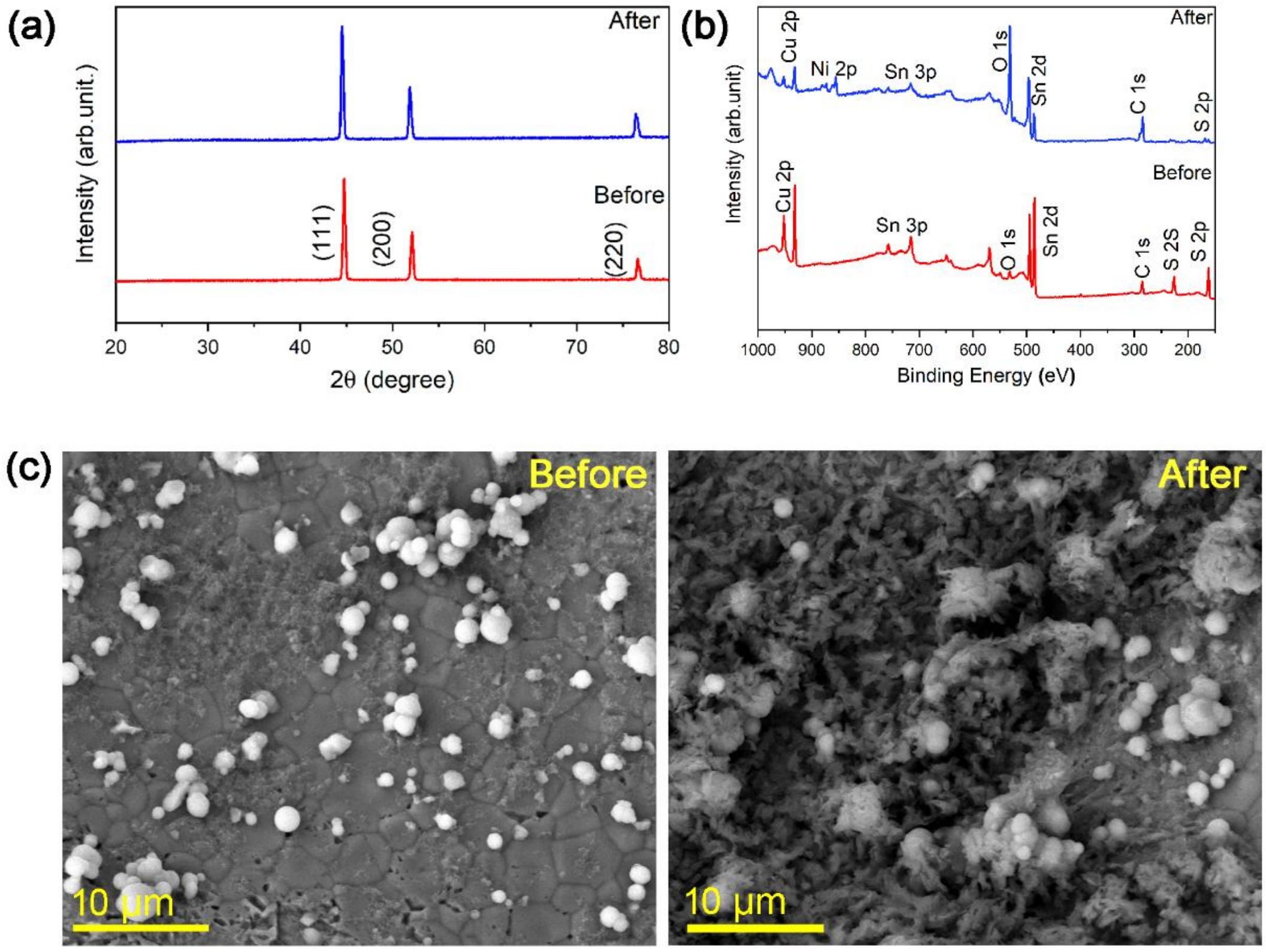


**Fig. S5:** (a) Typical XRD patterns, (b) XPS survey scans and (c) FESEM images of the CTS/Ni electrode before and after 24 h of HER electrochemical measurements.

The characteristic bright, near-spherical CTS features remain discernible in the FESEM images after prolonged electrocatalytic operation, indicating substantial retention of the original microstructural features and suggesting good morphological stability of the CTS.

## Supplementary Tables

**Table S1:** Reproducibility of the HER activity of independently prepared CTS/Ni electrodes

| Electrode | η at −10 mA cm$^{-2}$ (mV) | Tafel slope (mV dec$^{-1}$) |
|---|---|---|
| Sample 1 | 288 | 330 |
| Sample 2 | 288 | 330 |
| Sample 3 | 287 | 328 |
| Mean ± standard deviation | 288 ± 1 | 329 ± 1 |

**Overpotential is defined as** the potential required to achieve a current density of −10 mA cm$^{-2}$ . The very small SD indicates consistent measured values across the three electrodes.

**Table S2:** Comparison of the HER electrocatalytic performance of representative chalcogenide-based catalysts reported under neutral electrolyte conditions.

| **Catalyst** | **Electrolyte (pH7)** | **η (mV)** | **Tafel slope (mV dec$^{-1}$)** | **Reference** |
|---|---|---|---|---|
| Ni-$ZnIn_2S_4$/SS | 0.01 M PBS | - | 320–360 | 2 |
| $ZnIn_2S_4$/SS | 0.01 M PBS | - | >800 | |
| FeS | PBS | 700 | 150 | 3 |
| CZTS | 0.5M $Na_2SO_4$ | 690 | - | 4 |
| 17 % Co-$WS_2$ | 1M $Na_2SO_4$ | 514 | 90 | 5 |
| Ir-$WS_2$ | 1M $Na_2SO_4$ | 548 | 89 | 6 |
| Co–$Mo_{0.4}$–S nanoflowers | 1 M PBS | 213 | 94 | 7 |
| 3D $MoS_2$/N-doped graphene aerogel | 100 mM PBS | 261 | - | 8 |
| $MoSe_2$ based MSM/CC | 0.5 M $Na_2SO_4$ | 568 | 174.6 | 9 |
| 3D graphene/$CoS_x$ | 1 M PBS | 330 | 93 | 10 |
| $Co_9S_8$/C | PBS | 280 | - | |
| NiS/CC | PBS | 243 | 69 | |
| FeS | 0.1 M PBS | 350 | 150 | |
| $Cu_2SnS_3$ | 0.5M $Na_2SO_4$ | 288 | 330 | This work |

**References**


1. K. Jiang, B. Liu, M. Luo, S. Ning, M. Peng, Y. Zhao, Y. R. Lu, T. S. Chan, F. M. de Groot and Y. Tan, Nat. Commun., 2019, 10, 1743.
2. J. Lin, W. Li, Z. Zhao, D. Chen, X. Wang, J. Lu, Z. Ye and B. Lu, *Int. J. Hydrogen Energy*, 2024, **76**, 172–180.
3. E. M. Nichols, J. J. Gallagher, C. Liu, Y. Su, J. Resasco, Y. Yu, Y. Sun, P. Yang, M. C. Y. Chang and C. J. Chang, *Proc. Natl. Acad. Sci. U. S. A.*, 2015, **112**, 11461–11466.
4. R. V. Digraskar, B. B. Mulik, P. S. Walke, A. V. Ghule and B. R. Sathe, *Appl. Surf. Sci.*, 2017, **412**, 475–481.
5. A. Thennarasi and K. Vasu, *J. Power Sources*, 2025, **657**, 238241.
6. P. Siva and K. Vasu, *J. Mater. Chem. A*, 2024, **12**, 11101–11108.
7. L. Zhou, Z. Han, W. Li, W. Leng, Z. Yu and Z. Zhao, *J. Alloys Compd.*, 2020, **844**, 156108.
8. Y. Hou, B. Zhang, Z. Wen, S. Cui, X. Guo, Z. He and J. Chen, *J. Mater. Chem. A*, 2014, **2**, 13795–13800.
9. X. Peng, S. Xie, S. Xiong, R. Li, P. Wang, X. Zhang, Z. Liu, L. Hu, B. Gao, P. Kelly and P. K. Chu, *J. Energy Chem.*, 2023, **81**, 574–582.
10. S. Anantharaj, S. R. Ede, K. Sakthikumar, K. Karthick, S. Mishra and S. Kundu, *ACS Catal.*, 2016, **6**, 8069–8097.
